# Copper-binding proteins and exonic splicing enhancers and silencers

**DOI:** 10.1093/mtomcs/mfae023

**Published:** 2024-05-01

**Authors:** Dara Bakhtiar, Igor Vorechovsky

**Affiliations:** University of Southampton, Faculty of Medicine, Southampton SO16 6YD, UK; University of Southampton, Faculty of Medicine, Southampton SO16 6YD, UK

**Keywords:** metalloproteins, copper, Zn^2+^, Ca^2+^, the Irving–Williams series, alternative splicing

## Abstract

Eukaryotic DNA codes not only for proteins but contains a wealth of information required for accurate splicing of messenger RNA precursors and inclusion of constitutively or alternatively spliced exons in mature transcripts. This “auxiliary” splicing code has been characterized as exonic splicing enhancers and silencers (ESE and ESS). The exact interplay between protein and splicing codes is, however, poorly understood. Here, we show that exons encoding copper-coordinating amino acids in human cuproproteins lack ESEs and/or have an excess of ESSs, yet RNA sequencing and expressed sequence tags data show that they are more efficiently included in mature transcripts by the splicing machinery than average exons. Their largely constitutive inclusion in messenger RNA is facilitated by stronger splice sites, including polypyrimidine tracts, consistent with an important role of the surrounding intron architecture in ensuring high expression of metal-binding residues during evolution. ESE/ESS profiles of codons and entire exons that code for copper-coordinating residues were very similar to those encoding residues that coordinate zinc but markedly different from those that coordinate calcium. Together, these results reveal how the traditional and auxiliary splicing motifs responded to constraints of metal coordination in proteins.

## Introduction

Evolution of life on Earth involved a complex mixture of organic and inorganic compounds.^1^ Unlike enzymatically synthesized and degraded organic compounds, inorganic elements, once solubilized from the Earth's crust, are neither created nor destroyed, and must be under strict regulation to avoid metal toxicity and binding to organic molecules by noncognate metals (mismetalation).^1^ This regulation evolved over billions of years of life evolution, but is poorly understood, particularly in eukaryotic organisms with discrete and highly complex gene expression pathways.

Metal-binding complements of eukaryotic proteomes, or metalloproteins, require metal ions to assist catalysis or impart structure.^2^ In complex organisms, metalloproteins account for a large proportion of gene products,^3–6^ suggesting that evolution of metal interactions shaped not only protein coding but also regulatory components in eukaryotic DNA. For example, when considering splicing of precursor messenger RNA (pre-mRNA), the most complex gene expression step, human exons encoding coordinating residues for calcium (Ca^2+^) are richer in splicing enhancers as compared to exons that code for residues coordinating zinc (Zn^2+^).^7^ However, the lower capacity of “Zn^2+^ exons” to be included in mature transcripts can be compensated by intrinsically stronger splice sites, including stronger polypyrimidine tracts,^7^ key recognition motifs of splice acceptor sites of higher eukaryotes.^8,9^ Both exon groups showed higher than average exon inclusion levels in mature transcripts and a paucity of alternative splicing, ensuring efficient inclusion of codons for residues coordinating Ca^2+^ and Zn^2+^ in mature transcripts and their expression at the protein level.^7^ Although these data strongly suggest that the cells evolved to alleviate constraints imposed by metal coordination, responses of exon-intron architecture to coding restrictions imposed by other metals remain unknown.

Metalloproteins *in vitro* coordinate divalent metal ions with affinities largely reflective of the Irving–Williams series (Mn^2+^<Fe^2+^<Co^2+^<Ni^2+^<Cu^2+^ ≥ Zn^2+^). The series was first proposed by Harry Irving and Robert Williams who observed that the stability of complexes formed by divalent first-row transition metal ions generally increased across the period to a maximum stability at copper.^10–13^ The series was later proposed to include additional metals such as divalent *s*-block elements represented by weak alkali earth metals and was suggested to be an all-embracing principle for the control of metal ions in biology, balancing ion affinity and availability in the cell and predicting free metal concentrations for identical ligands.^11,12^ The series trend appears to operate within proteins as well as within small molecule chelators, contributing to their metal ion selectivity,^14^ but the extent of the Irving–Williams series universality is not completely understood.

Copper in cuproproteins is largely coordinated by the side chains of histidine, cysteine, and methionine^15^ and the frequencies of coordinating residues resemble those established for zinc.^16^ Because copper is the most competitive metal in the Irving–Williams series, human exons encoding Cu-coordinating residues would be expected to possess splicing silencer and enhancer profiles that are more similar to Zn^2+^ than to weaker metals, such as Mn^2+^ or alkali earth metals. However, it is not known if these residue preferences had any bearing on the “invisible” splicing code in exons and exonic splicing enhancers (ESE) vs. exonic splicing silencers (ESS) profiles during evolution. With the increasing number of cuproproteins described to date,^1,17^ we set out to test this question by comparing splicing enhancers and silencers in exons that encode Cu-coordinating residues with control exons and exons encoding binding sites for other metals. We have also determined their average inclusion levels in mature transcripts in human tissues and compared them to controls.

## Materials and methods

### Ascertainment of human exons that encode Cu-binding sites

We compiled (i) human proteins that were reported to bind copper, as supported by X-ray crystallography/solution nuclear magnetic resonance spectroscopy structures/electron microscopy and had Protein Data Bank (PDB) records (resolution lower than 3.6 Å). Residues were considered to coordinate copper if they were annotated in the PDB metal coordination tabs or specified as copper coordinating by the submitters in the supporting literature (Supplementary Table S1). Our sample of cuproproteins also included (ii) human proteins conserved between prokaryotes and humans, fungi and humans, or animals and humans if the copper-coordinating residues in PDB in lower organisms aligned with the same residue in human counterparts or with residues previously shown to coordinate this metal (Cys, His, Met, Tyr, Glu, Gly),^15^ with a few exceptions (*ATP7B, TYRP1, COMMD1, CTR1, LOXL2, XIAP*; Supplementary Table S1).^18^ Incomplete coordination spheres were included to maximize the codon pool size. Apart from PDB, the list of Cu-binding proteins was crosschecked against MetalPDB,^19^ Uniprot,^20^ and a previously published list of cuproproteins.^17^ Human gene symbols for the selected proteins were then matched to the Human Gene Nomenclature and Genecards records. The search yielded a set of 57 human proteins containing 491 unique codons for Cu-coordinating residues (December 2023; Supplementary Table S1). Finally, exons and adjacent introns encoding Cu-binding residues were retrieved from Ensembl^21^ and codons that encode Cu-coordinating residues (highlighted in red in Supplementary Table S1) and were used for ESE/ESS profiling as described.^7,22^

### Splicing enhancers and silencers for codons that encode Cu-coordinating residues

To characterize the auxiliary splicing code underlying Cu-binding sites in proteins, we employed a comprehensive set of human ESE and ESS hexamers derived by splicing promotion or repression *ex vivo*, which was measured for 4096 synthetic oligomers inserted into model *WT1* and *HBB* exons at five different positions.^23^ The resulting minigene libraries were used to acquire hexamer ESEseq and ESSseq scores defined previously.^23^ The scores were obtained independently of the presence or absence of metal-binding sites encoded by the test exons and provide good overall estimates of exon inclusion activities mediated by exon themselves.^23^ To assign ESEseq and ESSseq scores to Cu-coordinating codons, we computed average scores for overlapping hexamers using custom Microsoft Excel functions/formulas as described.^7^ To estimate codon-specific splicing activities, we also calculated frequency ratios for a total of 4728 ESE codons and 4360 ESS codons, presented here as *ln*(ESEf/ESSf), and we also determined codon counts in 1182 high-confidence ESEs and 1090 high-confidence ESSs^23^ to compute the ESEc/ESSc ratios, as previously defined.^7^ For control datasets, we extracted RefSeq sequences of human protein-coding exons as defined by the UCSC Table Browser (https://genome.ucsc.edu/cgi-bin/hgTables), comprising ∼35 million hexamers in ∼200 000 exonic segments.^7^ Apart from codons, mean ESEseq/ESSseq, ESEf/ESSf, and ESEc/ESSc values were also computed for entire exons that encode all Cu-coordinating residues. Exonic sequences were devoid of the first and the last three nucleotides (nts) since these exonic positions shape the 3′ss and 5′ss consensus, respectively.^24^

### Characterization of average mRNA inclusion levels

To determine PSI (% spliced in) values^25^ for tested exons, we employed PSI tables (hg38) from the Vertebrate Alternative Splicing and Transcription Database (VastDB), which provides comprehensive PSI values across vertebrate exons in various tissues and developmental stages.^26^ In addition, we compared PSI values of tested and control exons using expressed sequence tag (EST) data in HEXEvent, a database of Human EXon splicing Events, which stores EST-derived inclusion levels for ∼200 000 exons.^27^ HEXEvent shows additional alternative splicing information for human internal exons but unlike VastDB, HEXEvent does not include intron retention events, avoiding a bias toward short retained introns.^27,28^

## Results

### Auxiliary splicing code in exons that code for Cu-coordinating residues

To characterize ESE/ESS profiles of codons/exons that encode copper-binding function, we first obtained amino acid sequences surrounding copper-binding residues in 57 human cuproproteins (Supplementary Table S1). They were encoded by a total of 170 unique exons that contained 491 different codons for Cu-coordinating amino acids. Protein Cu-binding sites were dominated by His, Cys, and Met, in accordance with previous studies.^15^

**Fig. 1:**
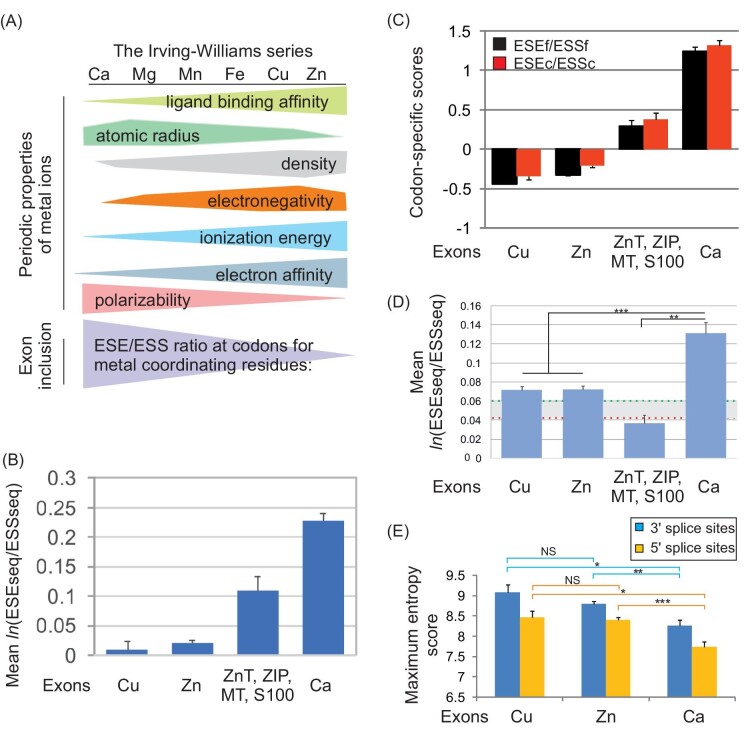
Splicing enhancers and silencers in exons that encode coordinating residues for copper, zinc, and calcium. (A) The Irving–Williams series and the auxiliary splicing code in exons. Upper panel shows selected properties of six abundant and biologically important divalent metals in the human body and their nonlinear trends in the Periodic Table of Elements. Atomic radius, the distance from atomic nucleus to outermost electron orbital; electronegativity, a tendency of an atom in a molecule to attract shared electrons; ionization energy, the amount of energy required to remove the first electron from neutral atoms; electron affinity, the energy released when an electron is added; and polarizability, tendency to acquire electric dipole moment in electric fields.^40^ Lower panel shows expected ESE/ESS profiles for codons encoding coordinating residues for the indicated metal in the series.^7,22,41^ (B, C) Estimates of exonic splicing enhancer and silencer strength for codons that encode coordinating residues for human cuproproteins (*n* = 57), zinc-binding proteins (*n* = 427, of which 293 were ZFs) excluding Zrt/Irt-like proteins, zinc transporters, metallothioneins and S100 (*n* = 55), calcium-binding EF-hand proteins (*n* = 189) and exon controls (horizontal bar) using ESEseq/ESSseq ratios (B) or codon-specific scores (C). The ESEseq/ESSseq values for controls were determined as previously described.^7,22^ (D) The mean ESEseq/ESSseq ratios in entire exons that encode coordinating residues for the same metals in the same protein groups. **P < 0.001, ***P < 0.0001. Horizontal bar denotes the range (maximum in green, minimum in red) of mean values for various control exon groups.^7,22^ (E) The intrinsic splice site strength of Cu, Zn, and Ca exons. *P < 0.01, **P < 0.001, ***P < 0.0001 (unpaired *t*-tests corrected for multiple comparisons). The sample of Cu exons had 146 and 153 unique 5′ and 3′splice sites, respectively. Error bars are SEMs Data for Zn ad Ca exons were taken from our previous study.^7,22^

We first assigned hexamer ESEseq and ESSseq scores to each codon and compared them to controls and those previously ascertained for human calcium- and zinc-binding proteins.^7,22^ For simplicity, we term these exons as Cu, Ca, and Zn exons. Figure 1B shows that ESEseq/ESSseq score ratios for Cu-coordinating codons resembled more Zn exons than Ca exons, as expected (see Introduction). The similarity was most pronounced for zinc finger proteins (ZFs) whereas other Zn-binding proteins, particularly those coordinated by acidic residues, showed higher values, albeit not reaching levels observed for enhancer-rich Ca codons (Fig. 1B and Bakhtiar *et al.*^7^) A similar pattern was found for codon-specific ESEf/ESSf and ESEc/ESSc scores (Fig. 1C). Taking into account extensions of codons close to 3′ or 5′splice sites to adjacent sequences (∼6% of the total codon count, Supplementary Table S1) yielded very similar values and the same order of tested metal exons.

We then asked whether the observed splicing dichotomy for two tight and one weak divalent metals of the Irving–Williams series can be seen at the level of entire exons. Figure 1D shows a comparison of mean ESEseq/ESSseq ratios for full exons that encode Zn-, Ca-, and Cu-coordinating residues and for control exons. Importantly, the capacity of ESE and ESS to include pre-mRNA segments encoding Cu-binding sites in mature transcripts was similar for Zn exons but significantly lower than for Ca exons, despite a smaller sample size.

Finally, we compared the intrinsic splice site strength of the studied metal exons (Fig. 1E) by computing maximum entropy scores for their 3′ and 5′ splice sites. We found that both 3′ and 5′ splice sites of Cu exons were similar to Zn exons, but significantly stronger than for the ESE-rich Ca exons. This was reflected by a higher number of uridines in the input sequence for maximum entropy scoring of 3′ splice sites [7.94 ± 0.32 (±SEM) for Cu exons, 8.16 ± 0.10 for Zn exons and 7.73 ± 0.18 for Ca exons], which provide rough estimates of the strength of polypyrimidine tracts.

Taken together, these results indicate the auxiliary splicing code of Cu exons is similar to Zn exons, but markedly weaker than for Ca exons as if recognition of exons that encode coordinating residues for tight metals in the Irving–Williams series (Zn, Cu) was selected against whereas that of exons encoding binding sites for weak Ca was encouraged in the cells to avoid mismetalation. They also show that this dichotomy was compensated by both 5′ and 3′ splice sites and their polypyrimidine tracts.

### Exon inclusion in mature transcripts and Cu binding in proteins

**Fig. 2:**
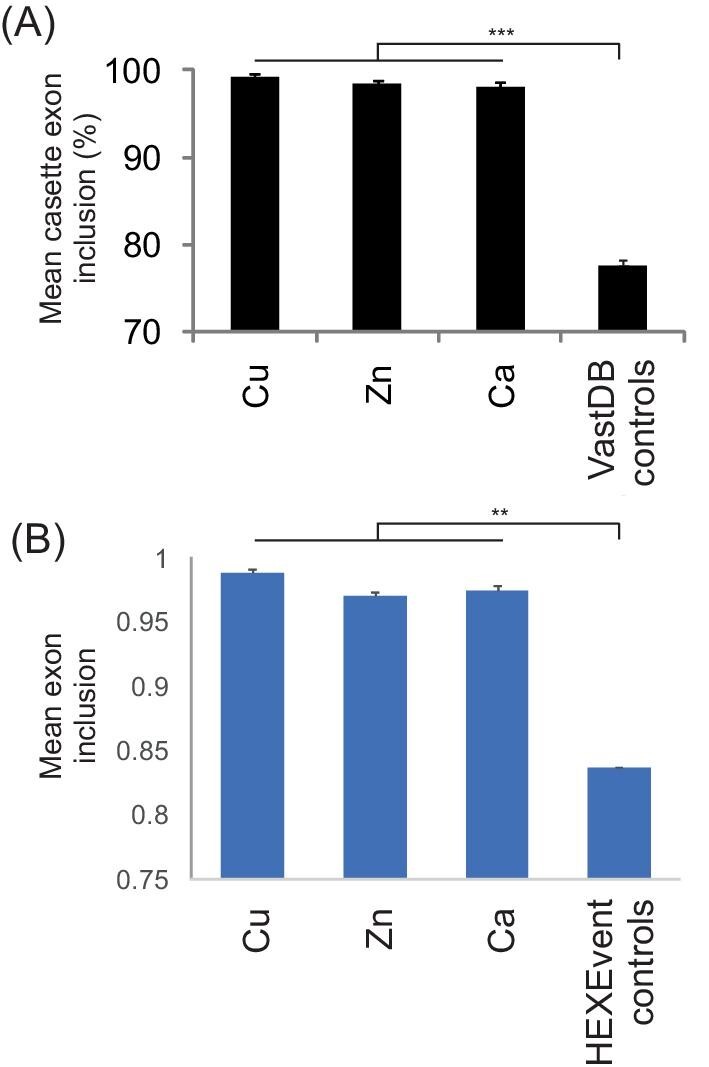
Mean *in vivo* inclusion of human exons that encode metal-coordinating residues in human proteins. (A) VastDB PSI values. (B) HEXEvent EST data. Error bars denote SEM, **P < 0.001, ***P < 0.0001.

**Fig. 3:**
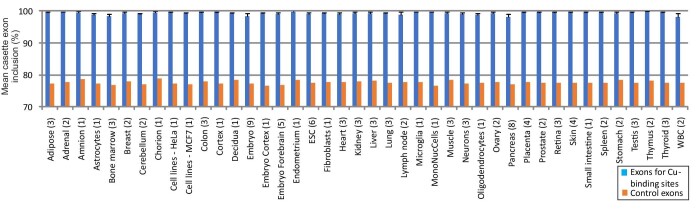
Average inclusion levels of Cu exons and VastDB controls in mature transcripts across multiple tissues. Number of independent tissue samples is shown in parentheses.

Next, we explored the question of whether the lower ESEseq/ESSseq profiles of Cu exons were reflected in lower exon inclusion levels *in vivo*; in other words, to what extent the observed dichotomous ESE/ESS profiles may influence their alternative splicing in the cell. We addressed the question by comparing average PSI values for Cu exons with control human exons and other groups of metal-coordinating exons previously analysed.^7,22^ Comparison of PSI values from the VastDB RNA-seq for Cu exons and control cassette exons showed that, similar to other exons that encode residues coordinating Zn and Ca,^7,22^ Cu exons had significantly higher exon inclusion levels (Fig. 2A). A similar difference was seen with HEXEvent EST data (Fig. 2B). The overall paucity of their alternative splicing was observed across multiple tissues (Fig. 3). We identified only a few Cu exons that showed PSI values below 80% in multiple tissues or repeated samples from the same tissue (Table 1). Although physiological roles of the potentially regulated splicing events and associated transcripts will require experimental verification, the overall high inclusion levels of Cu, Ca, and Zn exons (Fig. 2) are consistent with their critical role in metalloprotein expression.

**Table 1.** Selection of VastDB exons encoding copper-coordinating residues with low PSI values (<80%)

Cuproprotein	Gene symbol	Binding residue	VastDB exon ID	Exon size	Tissues
Copper-transporting ATPase β	*ATP7B*	C575	HsaEX0007040	162	Astrocytes, neurons, chorion
Hephaestin	*HEPH*	H965	HsaEX0029693	226	Embryonal forebrain, placenta pancreas, ovary, spleen
Synthesis of cytochrome C oxidase 1	*SCO1*	C169	HsaEX0056613	198	Embryo
Laccase domain containing 1	*LACC1*	G416	HsaEX0009320	161	Embryo, forebrain, kidney, liver
Coeruloplasmin	*CP*	H656	HsaEX0016927	213	ESCs
Amyloid β precursor like protein 1	*APLP1*	E362	HsaEX0005147	159	Heart, liver
Lysyl oxidase homolog 2	*LOXL2*	H626	HsaEX0036151	244	Pancreas

## Discussion

**Fig. 4:**
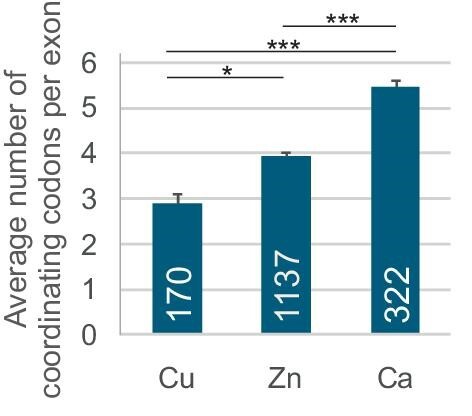
Mean number of codons for coordinating residues in Ca, Zn, and Cu exons. Number of exons is in white. Error bars denote SEM. Ca, codons coordinating Ca in binding loops of canonical EF-hand proteins. *P < 0.01, ***P < 0.0001, corrected unpaired *t*-test.

This study is the first to evaluate how copper-binding constraints in proteins influence the most complex gene expression step in eukaryotes. Eukaryotic exons are recognized by the largest RNA-protein complex in the cell with a single-nucleotide accuracy in the sea of much longer introns.^29^ To achieve this spectacular precision, the complex not only recognizes short motifs at splice sites but heavily relies on widespread ESEs, ESSs and additional intronic sequences in the pre-mRNA.^29^ Our study uncovers important differences in ESE/ESS profiles between Cu, Zn, and Ca exons, raising a speculation that the splicing machinery has not been immune to inorganic components of complex eukaryotes. However, coding restrictions that favor Zn and Cu binding to His/Cys codons and Ca binding to acidic residues could not be overcome by the “invisible” splicing code in exons. Their ESE/ESS profiles per se do not explain inclusion levels measured *in vivo*. Notably, the average density of coordinating codons in Cu, Zn, and Ca exons was not identical, with a hierarchy of Ca > Zn > Cu exons (Fig. 4), which is reminiscent of the Irving–Williams order. However, their density cannot explain ESE/ESS profiles. The correlation between codon counts and ESEseq/ESSseq ratios was poor if any [*r* = 0.14 (*P*-value = 0.03), 0.02 (*P* = 0.3) and 0.15 (*P* = 0.07) for Ca, Zn and Cu exons, respectively], consistent with only a marginal density contribution of coordinating residues in Ca exons coding for canonical EF hands.

Apart from exonic sequences, the constitutive inclusion of exons coding for weak and tight metals in mature transcripts (Fig. 3) is likely to require compensation by intronic splicing motifs, including stronger splice sites and polypyrimidine tracts.^7^ The tracts are highly variable traditional splicing recognition motifs that serve as more potent signals than ESEs/ESSs.^8,9^ Selecting mutated introns adjacent to Cu exons during evolution would be easier for the cell than selecting mutations around metal-binding residues, which may not be compatible with accurate splicing. It has become increasingly apparent that the cells face an incessant conflict between ensuring splicing efficiency and preserving the coding capacity for optimal proteins and their interactions.^30,31^ The fraction of synonymous mutations that affect RNA processing can be very high although it varies from exon to exon.^30,31^

Apart from chemical properties of metals themselves, biological utilization of metals has been influenced by geochemical pressures.^1,15,32^ Prokaryotes, particularly anaerobic bacteria and archaea, have adopted a very limited role for Cu, focusing largely on protection from Cu through sensing, intracellular mobilization and export.^1^ In contrast, the increasing mean intron per gene density in single-cell (0.1-∼1.5) and multi-cellular (up to 8 in mammals^33^) organisms has been associated with Cu delivery to expanding arrays of metalloproteins located within various organelles that facilitate tissue-specific regulation, indicative of tight control of Cu homeostasis both within and outside the cell.^1^ For example, the high-affinity Cu importer Ctr1 in *Saccharomyces cerevisiae*, which lacks introns, has evolved into a primary transporter *SLC31A1* (4 introns in zebrafish to humans) that is responsible for dietary Cu uptake in the intestinal epithelium. Studying evolution of introns surrounding metal-coordinating exons in more detail should provide deeper insights into the interplay of the “metal code” and traditional and auxiliary splicing signals.

Phylogenetic studies suggested that the protein folds for binding Mn and Fe evolved prior to the global oxygenation processes whereas the folds for Cu and Zn evolved after.^34^ Many cuproproteins have oxygen-related function, such as catalyzing redox reactions or dioxygen transport.^15^ The percentage of metal-binding sites within a proteome remains relatively stable over time, implying that the inclusion of a new metal is usually at the expense of another.^15,34^ It is still unclear, however, how released competitive ions (Cu and Zn) can target specific proteins in signaling pathways, such as Cu binding to ZFs that can alter their ZF function.^32,35–38^

Unlike Ca and Zn, Cu exists in oxidized (Cu^2+^) and reduced (Cu^+^) states in the cell. Free Cu is extremely limited within cells: the metal is highly toxic, particularly because of a high reactivity of Cu^2+^ and generation of OH radicals.^32^ Soluble Cu proved hazardous to early life, possibly by replacing other metal cofactors in proteins.^15^ As with oxygen, copper would eventually transform from poison to a necessity, with sophisticated networks for acquiring, transporting, sequestering, and exporting this metal.^15^ Copper proteins can traffic the metal or utilize it as a cofactor.^15^ The latter tend to use high-affinity sites of high coordination numbers that help prevent loss of the metal during redox reactions. The former, on the other hand, tend to use low-affinity sites with/or moderate coordination numbers.^15^ It was hypothesized that various combination of Cu-binding residues (His, Cys, Met) can afford dynamic multifunctional domains that can facilitate Cu transfer in various intra- and extracellular environments.^15^ For example, Cys may be more suitable for Cu accumulation in hypoxic environments where the metal is scarce whereas Met is well suited for Cu accumulation in acidic, oxidative milieu, with His preferred in neutral environments where Met-rich coordination spheres may not be sufficiently tight.^15^ Such preferences are likely to be reflected in ESE/ESS profiles that might in turn influence combinatorial diversity provided by the three ligands, which can offer a choice of neutral vs. charged environment, hydrophobic vs. hydrophilic, nitrogen vs. sulfur, and susceptibility to pH changes or oxidation.^15^

The number of cuproproteins in our study was comparable to a recent count,^17^ but their final number in human cells is not yet known. The sample size of Cu exons had a smaller statistical power compared to Zn or Ca exons, preventing us to address intronic contributions in more detail or compare the three abundant metals, as accomplished for Zn^2+^ and Ca^2+^.^7^ The excess of Cu^2+^ over Cu^+^ in our study was by a factor of ∼3, hampering detailed ESE/ESS comparisons of coordination spheres for cupric and cuprous ions, which may vary with oxidation states. It is not yet known which alternative splicing events control relatively high copper concentrations in copper storage vesicles that have been identified in the brain and other cell types^39^ and how they are regulated at the RNA level. Not in the least, the identification of physiological roles of metals remains to be technically challenging, and has been hindered by examples of ion dissociation during protein or nucleic acid purification, limited detection in structural models, the impact of overexpressing metal-binding molecules on metal availability, and metal interchangeability.^32^ Some of these questions may attract new investigators in the emerging field of alternative RNA processing in metal signaling.

## Supplementary Material

mfae023_Supplemental_File

## Data Availability

The data underlying this article are available in the article in its online supplementary material.
